# Improving p*K*_a_ Predictions with Reparameterized Force Fields and Free Energy Calculations

**DOI:** 10.1021/acs.jctc.5c00031

**Published:** 2025-04-02

**Authors:** Carter J. Wilson, Vytautas Gapsys, Bert L. de Groot

**Affiliations:** † Computational Biomolecular Dynamics Group, Max Planck Institute for Multidisciplinary Sciences, Göttingen 37077, Germany; ‡ Computational Chemistry, Janssen Research & Development, Janssen Pharmaceutica N. V., Turnhoutseweg 30, Beerse B-2340, Belgium

## Abstract

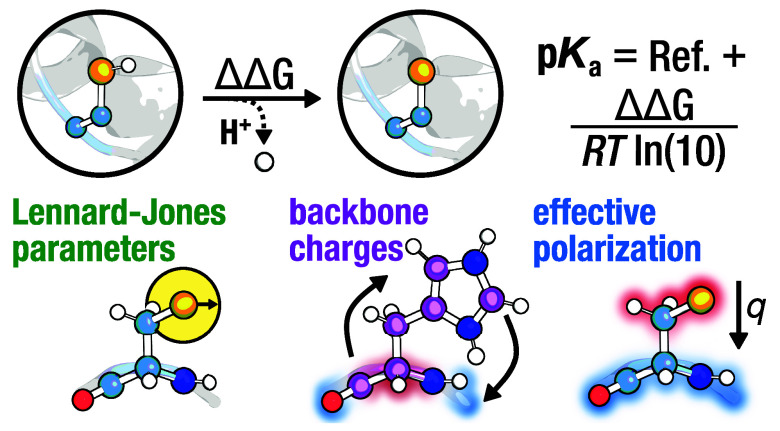

Given the growing interest in designing targeted covalent inhibitors, methods for rapidly and accurately probing p*K*_a_s—and, by extension, the reactivities—of target cysteines are highly desirable. Complementary to cysteine, histidine is similarly relevant due to its frequent presence in protein active sites and its unique ability to exist in two tautomeric states. Here, we demonstrate that nonequilibrium free energy calculations can accurately determine the p*K*_a_ values of both residues, often outperforming conventional predictors. Importantly, we find that (1) increasing the van der Waals radius of cysteine’s sulfur atom, (2) modifying the backbone charges of histidine, and (3) introducing effective polarization by downscaling the side chain partial charges of both residues can all significantly improve p*K*_a_ prediction accuracy. Using the modified CHARMM36m force field on the full dataset reduces the prediction error from 2.12 ± 0.27 p*K* to 1.28 ± 0.15 p*K* and increases the correlation with experiment from 0.25 ± 0.09 to 0.58 ± 0.08. Similarly, using the modified Amber14SB force field decreases the error from 3.21 ± 0.29 p*K* to 1.69 ± 0.23 p*K* and improves the correlation from 0.15 ± 0.10 to 0.36 ± 0.10.

## Introduction

Cysteine is unique among the 20 proteogenic amino acids due to the large atomic radius of its sulfur atom and the relative weakness of the corresponding S–H bond. This imbues it with remarkable nucleophilicity, facilitating spontaneous reactions even under mild conditions.^1^ The inherent nucleophilicity of a given cysteine is governed by its p*K*_a_, a value that implies the favorability of the ionization state of the thiol. Solvent-exposed cysteines have values near 8,^2,3^ while buried cysteines or those located in a unique protein microenvironment can range from 3 to 12.^4,5^ Given their variable p*K*_a_ values and unique properties, cysteine residues play various functional roles in redox and nucleophilic catalysis,^6^ metal binding,^7^ environmental sensing,^8^ and structural formation.^9,10^

In recent years, interest has grown in targeting cysteine residues with covalent inhibitors.^11^ To overcome poor target selectivity and drug resistance, an electrophilic warhead moiety may be incorporated into a reversible submicromolar inhibitor to covalently bind a nucleophilic residue: this modification can dramatically increase therapeutic potency.^12,13^ Members of this class of reactive molecules are commonly referred to as targeted covalent inhibitors (TCIs) and predicting their affinity and reversibility is particularly desirable.^14^

A key step in a TCI binding and reaction landscape is the deprotonation of the cysteine thiol and the formation of the nucleophilic thiolate. Experimental exchange-rate studies have shown that the equilibrium between the protonated and deprotonated states of solvent-exposed cysteine side chains is fast^15^ (i.e., 10^12^· *M*^–1^*s*^–1^) and that the protonation rate and p*K*_a_ are well correlated.^15,16^ That is to say, the p*K*_a_ of a particular cysteine provides the relevant information about the energy required to form the nucleophilic thiolate and, by extension, the propensity for covalent modification.

**Figure 1:**
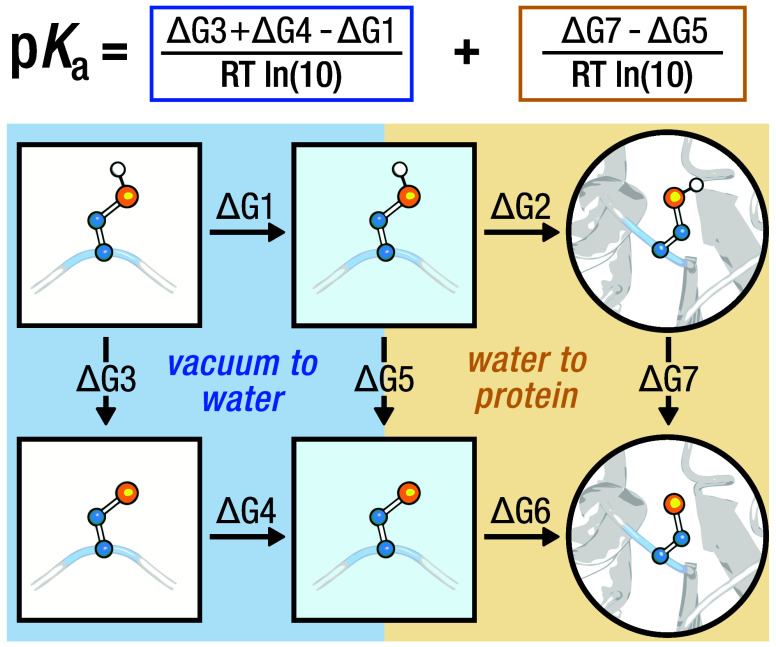
Complete p*K*_a_thermodynamic cycle. The horizontal arrows mark the transfer of a titratable residue between different environments: vacuum (left), water (middle), protein (right). The vertical arrows denote the free energy difference between the deprotonated and protonated form in a corresponding environment. For cysteine, the free energy associated with proton transfer from vacuum to water is known. By using this reference  we need only consider the rightmost cycle: the free energy difference of a deprotonation event in water and in protein.

Experimental methods for determining the p*K*_a_ value of a cysteine can involve kinetic assays, spectrophotometric titrations, or NMR spectroscopy; however, in a purely computational *in silico* screen of potential covalent modifiers, the ability to rapidly and accurately probe the reactivity of a target cysteine under various conditions is highly desirable. Theoretical approaches motivated by the thermodynamic cycle given in Figure 1, present a compelling alternative to experiment and can often be seamlessly integrated alongside existing computational free energy workflows.

Here, we consider a cysteine residue in the protein and a capped model peptide (i.e., ACE-Ala-Cys-Ala-NH_2_) in both vacuum and water. We have the reference  and as such can neglect the free energy of moving a (de)protonated residue from vacuum to water (Figure 1, left cycle). To determine the p*K*_a_ we then need only consider the free energy difference of a deprotonation event in water and in the protein (Figure 1, right cycle).

Recently we demonstrated that atomistic molecular dynamics simulations paired with nonequilibrium alchemical free energy calculations are capable of accurately resolving the p*K*_a_ values of aspartate, glutamate, and lysine.^17^ Here, we assess the ability of our pmx-based, nonequilibrium switching (NES) approach to calculate the p*K*_a_ values of 40 cysteines and 22 histidines across a range of wildtype and mutant proteins. We compare our results to two conventional predictor methods and the previously reported performance of three MD-based approaches, replica-exchange thermodynamic integration, constant-pH molecular dynamics, and free energy perturbation. Taken as a whole, our results demonstrate that MD-approaches, including NES, provide p*K*_a_ prediction accuracy comparable to conventional predictors; however, we also find that this accuracy can be increased well above conventional methods by employing parameters that are refit to more accurately reproduce QM and experimental observables, i.e., (1) rescaling the vdW radii of cysteine sulfur thiolate and (2) altering the backbone charges of histidine in Amber14SB, and (3) charge-scaling CHARMM36m and Amber14SB. Using the triple-modified Amber14SB force field we achieve an average unsigned error of 2.37 ± 0.29 p*K* for cysteine and 0.50 ± 0.10 p*K* for histidine, whereas using charge-scaled CHARMM36m, we achieve errors of 1.61 ± 0.21 p*K* for cysteine and 0.71 ± 0.16 p*K* for histidine.

## Methodology

### Cysteine Analogues: QM Simulations

*Ab initio* molecular dynamics (AIMD) simulations were performed within the Born–Oppenheimer approximation; where the electronic structure of the system is solved using the Gaussian plane-wave (GPW) approach to DFT,^18,19^ implemented in the QUICKSTEP^20^ subroutine of CP2K.^21^ We used the standard LIBXC library^22^ for the exchange and correlation of the revPBE functional^23,24^ and applied Grimme’s DFT-D3 dispersion corrections with zero-damping.^25^ We use the Goedecker–Teter–Hutter pseudopotentials^26,27^ optimized for PBE to represent the core electrons and the TZV2P basis set. Simulations consisted of an initial 10 ps equilibration, followed by a production run in the NVT ensemble for another 200 ps. The temperature was maintained at 298 K by a massive Nose-Hoover chain thermostat with a time constant of 3 ps. We set an energy convergence threshold of 10^–10^ Ha and a convergence tolerance for the SCF cycle of 10^–6^ Ha. Because the *ab initio* simulations were to be performed in the NVT ensemble, achieving an accurate initial box volume was important. To this end, we performed classical molecular dynamics implemented in GROMACS with the OPC water model to produce an initial system configuration. OPC was chosen because it more accurately reproduces the relevant bulk properties of water compared to conventional 3-point models (i.e., TIP3P).^28^ The result of these classical simulations was a final cubic box size of *L* = 15 Å, containing 109 water molecules and a methylthiolate molecule.

### Cysteine Analogues: MD Simulations

Classical MD simulations were performed in three types of boxes: one identical to that used in AIMD simulations (i.e., *L* = 15 Å) and two larger boxes: *L* = 30 Å and *L* = 60 Å (Figure S1). We found no significant differences in the solvation structure when comparing the 15 and 30 Å boxes (Figure S3a). This observation was independent of the cutoff used for the simulation of the larger box (Figure S4).

Solvation free energy calculations of the charged methylthiolate molecule were performed in both a 30 and 60 Å box. Importantly, we found no change in the calculated free energy between the two box sizes (Figure S5a) which suggests the 30 Å box is sufficiently large to minimize the non-neutral simulation cell artifact associated with the calculation.

GROMACS 2023^29^ was used to run all simulations. Simulations were carried out in the NVT ensemble with a constant temperature of 298 K, maintained using a Nosé–Hoover thermostat with 3 ps coupling time. In the 30 and 60 Å boxes, long-range electrostatic interactions were calculated using the Particle-Mesh Ewald method^30^ with a real-space cutoff of 1.2 nm and grid spacing of 0.12 nm with CHARMM and a real-space cutoff of 1.0 nm and grid spacing of 0.125 nm with Amber. For CHARMM the Lennard-Jones interactions were force-switched off between 1.0 and 1.2 nm, while for Amber, a cutoff at 1.0 nm was used and a dispersion correction was applied to the energy and pressure.

In the 15 Å box, electrostatic interaction cutoffs were 0.7 nm with a 0.12 nm spacing with CHARMM and 0.7 nm with a 0.125 nm spacing with Amber. For CHARMM the Lennard-Jones interactions were force switched off between 0.5 and 0.7 nm, while for Amber they were cut off at 0.7 nm and a dispersion correction was applied to the energy and pressure.

We used the CHARMM TIP3P water model with the nonzero Lennard-Jones parameters on hydrogen atoms (i.e., mTIP3P)^31^ and plain TIP3P^32^ for the CHARMM and Amber systems, respectively. In the case of CHARMM we also assessed the impact of using the OPC water model on the solvation structure and measured free energies.

Production simulations were 50 ns and the first 10 ns of simulation were discarded as equilibration. From the remaining 40 ns: (1) radial distribution functions were computed; and (2) 200 nonequilibrium transitions of 20 ps were generated. Free energies were computed as described in the final paragraph of the following section.

### GROMACS/pmx: Nonequilibrium Alchemy

pmx(33) was used for system setup, hybrid topology generation, and analysis. Initial protein structures were taken from the PDB database and mutations were introduced using pdbfixer. In total we consider 12 proteins and 40 cysteine residues and 22 histidines (Table S1). A double system in a single box setup was used, with a 3 nm distance between the protein and peptide (ACE-Ala-X-Ala-NH_2_); this ensured a neutral box at every step of the alchemical transformation. To ensure that the protein and peptide did not interact, a single Cα in each molecule was positionally restrained. We used the CHARMM36m^34^ (with mTIP3P^31^) and Amber14SB^35^ (with TIP3P^32^) force fields, both having previously performed well for simulations involving nonequilibrium alchemical calculations.

GROMACS 2023 was used to run all simulations. For all systems, an initial minimization was performed using the steepest descent algorithm. A constant temperature corresponding to the reference experimental setup was maintained implicitly using the leapfrog stochastic dynamics integrator^36,37^ with a friction constant of γ = 0.5 ps^–1^. The pressure was maintained at 1 bar using the Parrinello–Rahman barostat^38^ with a coupling time constant of 5 ps. The integration time step was set to 2 fs. Long-range electrostatic interactions were calculated using the Particle-Mesh Ewald method^30^ with a real-space cutoff of 1.2 nm and grid spacing of 0.12 nm. Lennard-Jones interactions were force-switched off between 1.0 and 1.2 nm. Bonds to hydrogen atoms were constrained using the Parallel LINear Constraint Solver.^39^

Production simulations were 50 ns in length and run in quadruplicate. The first 10 ns of simulation was discarded as equilibration and from the remaining 40 ns, 400 nonequilibrium transitions of 200 ps were generated. Work values from the forward and backward transitions were collected using thermodynamic integration and these were used to estimate the corresponding free energy with Bennett’s acceptance ratio^40^ as a maximum likelihood estimator relying on the Crooks Fluctuation Theorem.^41^ Bootstrapping was used to estimate the uncertainties of the free energy estimates, and these were propagated when calculating ΔΔ*G* values.

As shown in Figure 1, we convert between the ΔΔ*G* of deprotonation and the protein p*K*_a_ via:
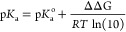
1using the experimentally reported temperature and corresponding reference values for cysteine  and histidine .^3^

### Conventional Predictors

Based on popularity and previously documented cysteine p*K*_a_ prediction performance^42^ we considered only two methods: Prop*K*_a_ (v3.4)^43^ and Pyp*K*_a_ (v2.9.4).^44^ Prop*K*_a_ is an empirical predictor where the Δ*G* contributions are described by charge–charge, desolvation, and hydrogen-bonding interactions. Default settings were used when performing the calculation. Pyp*K*_a_ uses Monte Carlo simulations to probe the various side chain states and employs DelPhi^45^ to resolve the PBE. Default settings were used, except for the salt concentration, which was set according to the experimental setup.

Recently, Molecular Operating Environment (MOE) was used to calculate the p*K*_a_ values of a large cysteine residue data set.^42^ We compare NES with this method on the overlapping 20 residue data set.

## Results

### Overall Performance: Original Force Fields

Double free energy differences (ΔΔ*G*) were calculated for a set of 40 residues, allowing us to robustly evaluate performance on a large data set.

**Figure 2:**
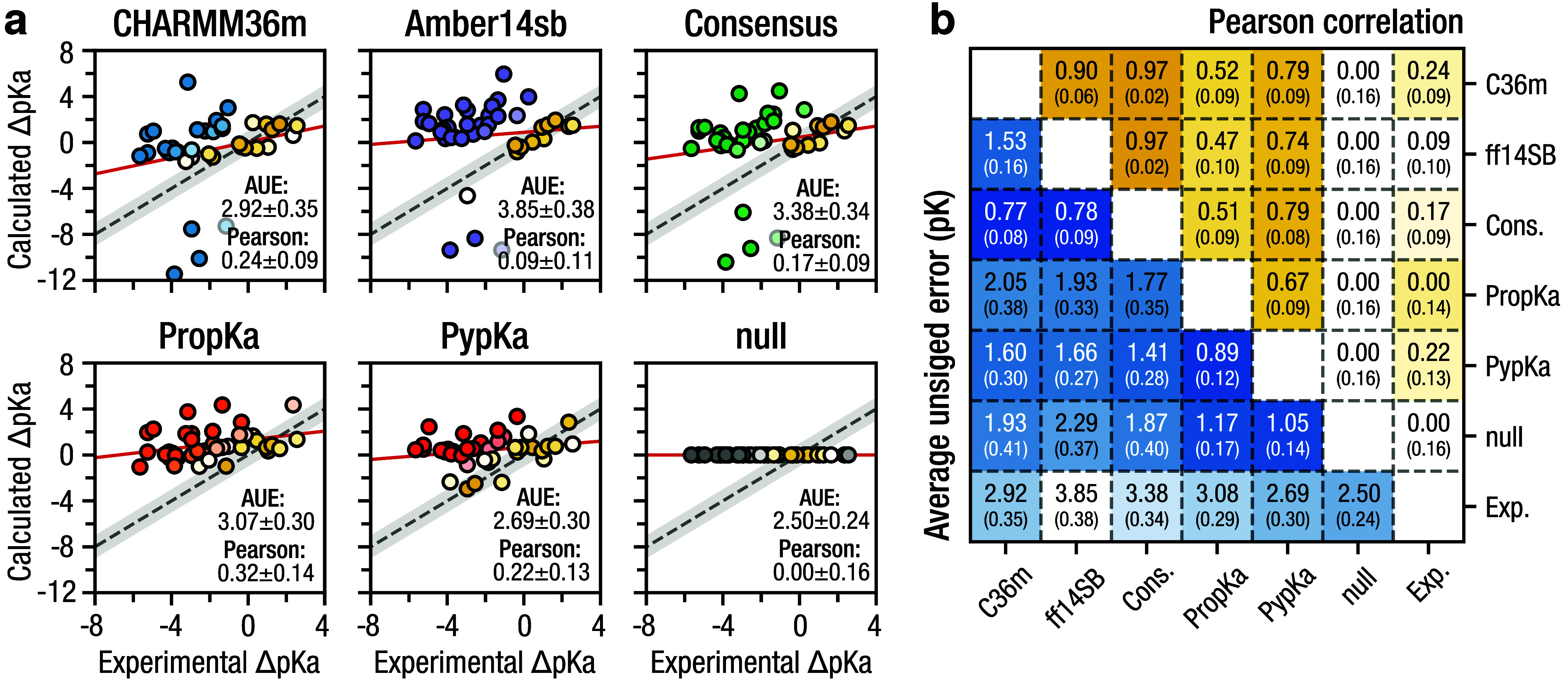
Overall performance: original force fields and predictors. (a) Correlation between the calculated and experimental cysteine p*K*_a_ values. Marker color indicates deviation from experiment where yellow indicates a minimum AUE from experiment. Regression lines are shown in red, and the gray error band represents a 1 p*K* unit deviation from experiment. Consensus is the average of CHARMM36m and Amber14SB. (b) Pearson correlations (upper right triangle) and AUEs (lower left triangle) between Δp*K*_a_ estimates were calculated for each method over the entire data set. Comparison with experiment means that the bottom row and rightmost column correspond to the overall performance.

Figure 2 summarizes the main findings: our NES approach performs comparably to *in silico* predictors, with CHARMM36m yielding an average unsigned error (AUE) of 2.92 ± 0.35 p*K* as compared to 3.09 ± 0.29 p*K* and 2.71 ± 0.29 p*K* for Prop*K*_a_ and Pyp*K*_a_, respectively. This performance was also reflected in the Pearson correlation, which was 0.24 ± 0.09 with CHARMM36m compared to 0.31 ± 0.14 and 0.22 ± 0.12 with Prop*K*_a_ and Pyp*K*_a_, respectively. On the 20 residue subset evaluated by MOE,^42^ MOE exhibited an improved accuracy of 1.79 ± 0.24 p*K* as compared to 2.49 ± 0.46 p*K* and 2.95 ± 0.45 p*K* for CHARMM36m and Amber14SB, respectively. With respect to accuracy, no method significantly exceeds a null predictor, which assumes Δp*K*_a_ = 0.

Previous work has illustrated that an accurate determination of the p*K*_a_ may require accounting for residue coupling.^46^ With respect to cysteine residues, often found at enzyme active sites, the relevance of coupling is expected to become even more pronounced. Elsewhere, we introduced a coupling formalism that improved p*K*_a_ prediction accuracy;^46^ here, we apply this approach to 12 cysteine residues found near other titratable groups. Consistent with previous work, the p*K*_a_ values of coupled residues were predicted with lower accuracy, when the coupling was not explicitly accounted for. Accounting for coupling, however, could in part remedy this (Figure S2). The observed improvement was less pronounced for Amber14SB (AUE without coupling: 3.85 ± 0.38 p*K*, AUE with coupling: 3.53 ± 0.37 p*K*) compared to CHARMM36m (AUE without coupling: 2.92 ± 0.35 p*K*, AUE with coupling: 2.28 ± 0.28 p*K*). On the 20 residue subset evaluated with MOE, accounting for coupling was even more pronounced for CHARMM36m, shifting the accuracy from 2.49 ± 0.46 p*K* to 1.53 ± 0.29 p*K*.

Previous work has assessed the ability of different MD-based approaches to predict cysteine p*K*_a_ values^42,47^ we can compare our performance on the overlapping data sets. For 18 cysteine residues, Awoonor-Williams and Rowley,^47^ found a thermodynamic integration, replica-exchange scheme with the CHARMM36 force field gave an AUE of 1.67 ± 0.40 p*K* (compared to 1.64 ± 0.40 p*K* with CHARMM36m and NES). More recently, Awoonor-Williams and coworkers^42^ found that on 25 residues, a Monte Carlo, constant-pH approach paired with CHARMM36m gave an AUE of 2.42 ± 0.36 p*K* (compared to 1.70 ± 0.34 p*K* with CHARMM36m and NES).

Consistent with previous work, CHARMM36m performed significantly better than plain Amber14SB (Figure 2); this large discrepancy led us to investigate the underlying parameterization differences.

### Thiolate Reparameterization: Amber14SB-1.3σ

In the Amber family of force fields, both the thiol and thiolate sulfur atoms share the same atom type, while the partial charge assignments between the two residues differ. Thiolate sulfur has a more diffuse electron density and a larger ionic radius, characteristics that will be reflected in the Lennard-Jones parameters, particularly the σ-value.

**Figure 3:**
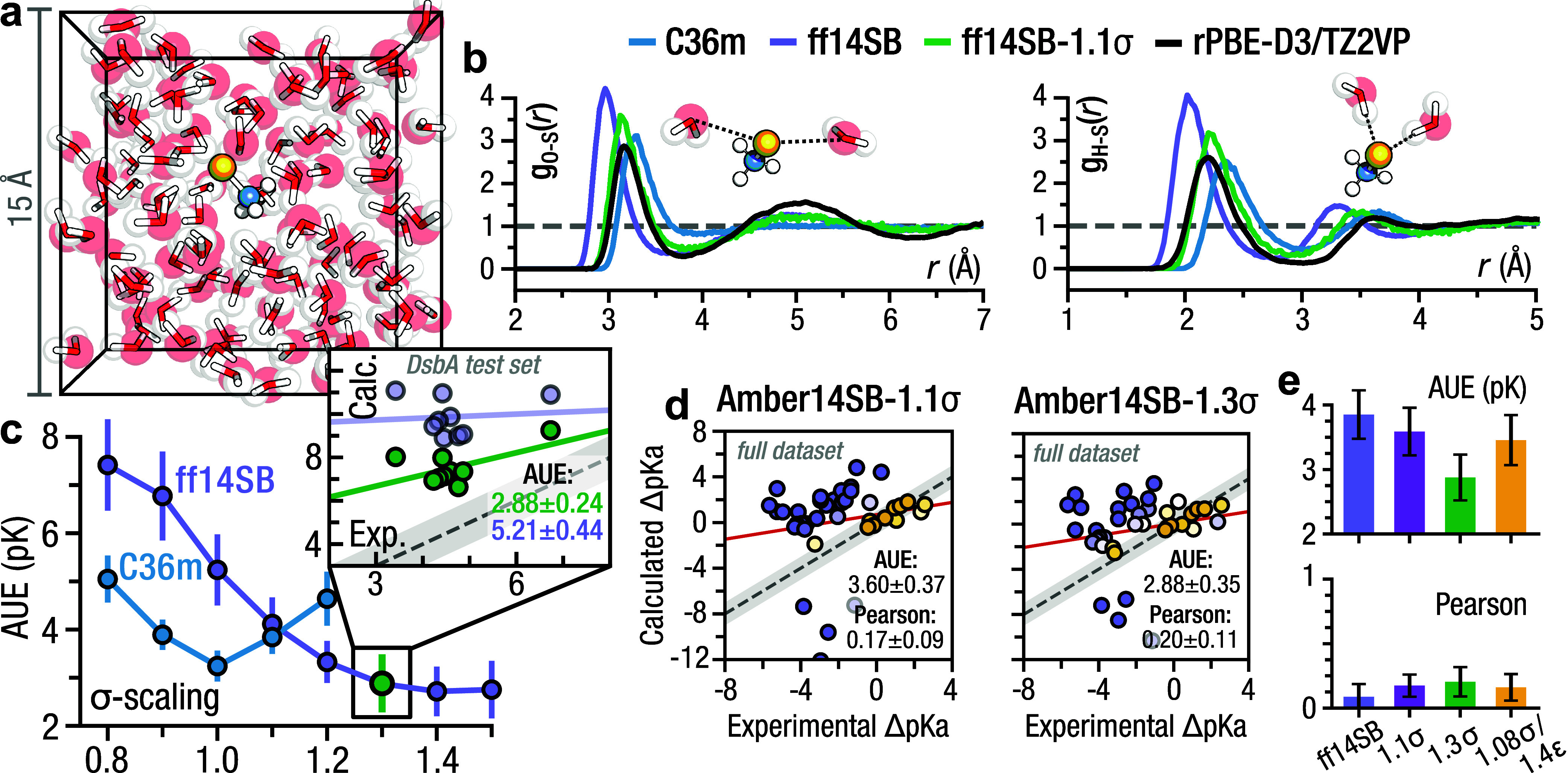
Thiolate reparameterization: Amber14SB-1.3σ. (a) *Ab initio* simulation setup with methylthiolate and ≈100 water molecules. (b) Radial distribution function between the methylthiolate sulfur and water oxygen (left) and methylthiolate sulfur and water hydrogen (right). The *ab initio* distribution is shown as a black line. (c) p*K*_a_ prediction performance for CHARMM36m and Amber14SB on the DsbA test set as a function of σ-value scaling. Amber14SB-1.3σ performance is marked in green. (c, inset) Correlation between the calculated and experimental p*K*_a_ values on the DsbA test set, with regression lines indicated. The gray error band represents a 1 p*K* unit deviation from experiment. (d) Correlation between the calculated and experimental cysteine p*K*_a_ values. Marker color indicates deviation from experiment where yellow indicates a minimum AUE from experiment. Regression lines are shown in red, and the gray error band represents a 1 p*K* unit deviation from experiment. (e) Prediction performance across the full data set, comparing the scaled Amber14SB force fields.

Simulations of methylthiolate using both classical molecular dynamics with Amber14SB and CHARMM36m, as well as *ab initio* molecular dynamics, revealed substantially different hydration structures (Figure 3a,b). Specifically, Amber14SB methylthiolate exhibited a radial distribution function (RDF) peak backshift of 0.5 nm compared to AIMD, suggesting a potentially erroneous hydration structure (Figure 3b). We note that while the revPBE-D3 functional has previously been shown to well reproduce the solvation structures of water^48^ and anions like chloride^49,50^ as a pure GGA functional it has a tendency to overdelocalize electrons, which may shift the first peak position of *g*(*r*) to a larger distance. Importantly, the position of the first peak in our O–S RDF, i.e., *r* ≈ 3.16 Å is consistent with that observed in two separate AIMD studies of methylthiolate solvation which calculated values of *r* ≈ 3.20 Å and *r* ≈ 3.10 Å, employing higher-level hybrid and range-separated functionals.^47,51^

Given the significant discrepancy in prediction performance between CHARMM36m and Amber14SB, as well as the notable differences in RDFs, we rescaled the Lennard-Jones σ value to improve agreement with the AIMD RDF and potentially improve p*K*_a_ prediction performance.

Exploring the matrix of rescaled σ- and ϵ-values within the interval [0.5, 1.5] with 0.1 spacing, we found that a σ-value of 1.1 well reproduced the oxygen–sulfur RDF from the AIMD trajectories (Figure 3b). Expanding the grid to include more values yielded the same conclusion. Because adjusting the ϵ-value led to only marginal enhancements (Figure S3b), we refrained from unnecessarily fitting both parameters.

We performed a similar analysis to determine an optimal value for predicting the solvation free energy of methylthiolate.^52^ Consistent with the RDF analysis, we observed that modifications to σ yielded more significant improvements than changes to ϵ (Figure S5b); however, achieving an accurate solvation-free energy required a σ-scaling of 1.3 (Figure S5a).

With the primary goal of improving p*K*_a_ prediction performance we probed the p*K*_a_ values of wild type DsbA and seven mutants. Because changes in ϵ had a limited effect on solvent structure or solvation free energy, we decided to only scan σ-values on the interval [0.8, 1.5]. We observed a sigmoidal improvement in accuracy that was saturated for σ = 1.3 with an AUE of 2.88 ± 0.24 p*K* (Figure 3c) and a Pearson correlation of 0.31 ± 0.60. This accuracy was significantly increased from unscaled Amber14SB which had an AUE of 5.21 ± 0.44 p*K* and a correlation of 0.00 ± 0.51 on the DsbA test set.

Using Amber14SB-1.3σ on the full data set gave an AUE of 2.88 ± 0.35 p*K* and a Pearson correlation of 0.20 ± 0.11 (Figure 3d): markedly improved from the performance of plain Amber14SB, but still worse than the accuracy previously reported for aspartate, glutamate, and lysine. Using the σ-value that maximized agreement with the *ab initio* determined solvation structure (i.e., σ = 1.1), yielded an AUE of 3.60 ± 0.47 p*K* and a Pearson correlation of 0.17 ± 0.09 (Figure 3d). Previous efforts to reparameterize the thiolate parameters for cysteine targeted both the σ- and ϵ-values.^51^ Optimizing against the AIMD solvation structure of methylthiolate, the researchers found a scaling factor of 1.08 for σ and 1.40 for ϵ provided the best agreement. Using these parameters we observed a statistically significant AUE improvement over the original Amber14SB of 0.38 p*K* (Figure 3e, Figure S7) which is comparable to the ≈0.5 p*K* improvement reported using the same parameters with Amber99SB on a different data set.^42^ As discussed above, scaling σ by 1.1, which best matched our AIMD solvation structure, resulted in a nonsignificant AUE improvement of 0.25 p*K*, while further scaling to 1.3 did yield a significant improvement of 0.97 p*K* (Figure 3e).

We performed an identical analysis for CHARMM36m, which indicated that although a larger σ value (i.e., σ ≈ 1.15) was required to achieve an accurate experimental solvation free energy (Figure S5a), the default LJ parameters could effectively reproduce the RDF data (Figure 3b) and maximize p*K*_a_ prediction accuracy on the DsbA test set (Figure 3c); this observation led us to leave the σ parameter untouched. Using the OPC water model (rather than mTIP3P) resulted in an identical position of the first solvation shell (Figure S6a) and suggested the same scaling factor was required to reproduce the experimental solvation free energy (Figure S6b).

In short, the default Amber14SB cysteine thiolate parameters are erroneous and increasing σ or both σ and ϵ is required to better reproduce QM and experimental observables. Nevertheless, even with this modification, CHARMM36m still exceeds the accuracy of Amber14SB–1.3σ.

### Histidine Partial Charges: Amber14SB-H

**Figure 4:**
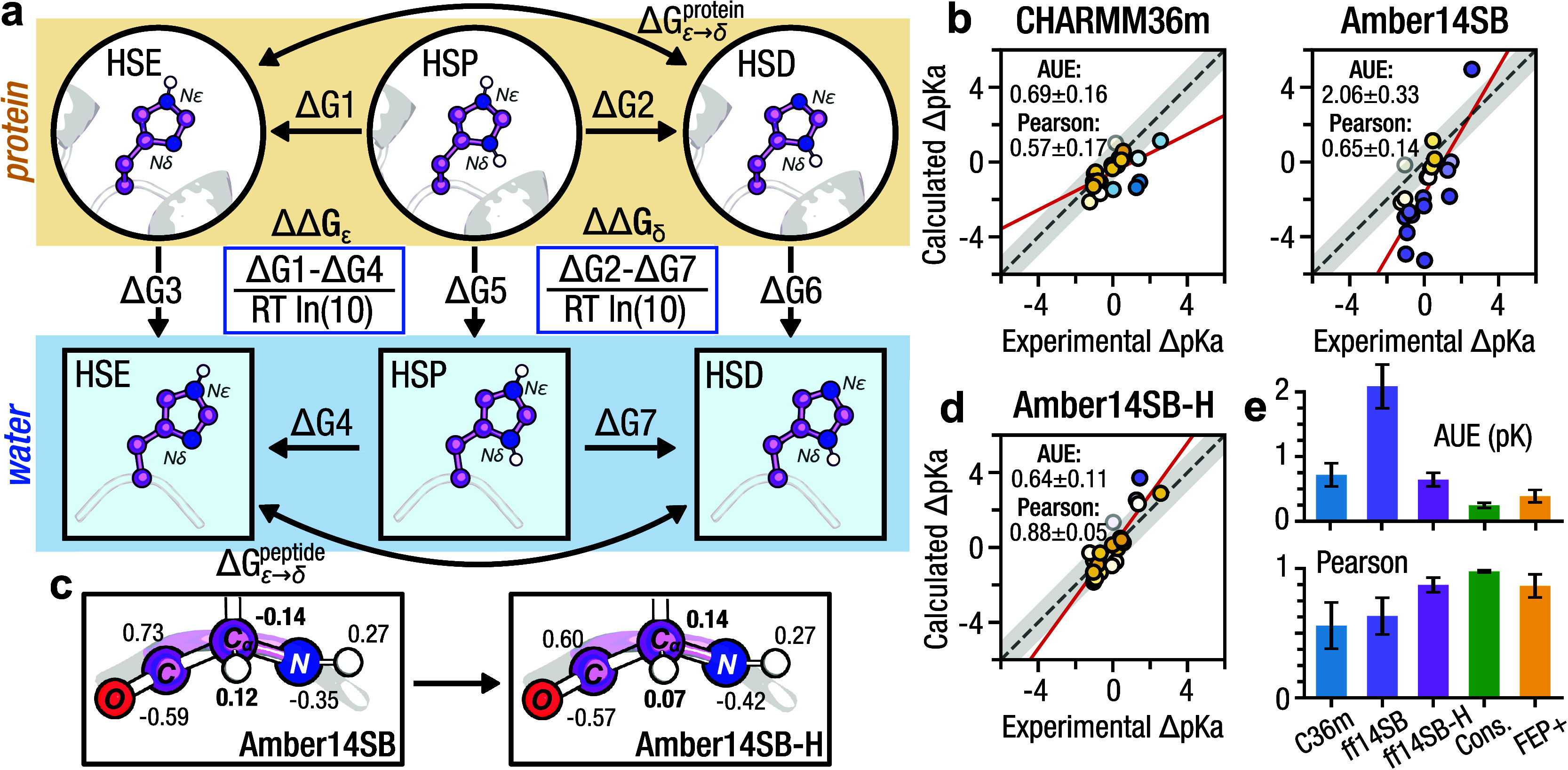
Histidine partial charges: Amber14SB-H. (a) Thermodynamic cycle corresponding to the p*K*_a_ of histidine. (b) Correlation between the calculated and experimental histidine p*K*_a_ values. Marker color indicates deviation from experiment where yellow indicates a minimum AUE from experiment. Regression lines are indicated in red, and the gray error band represents a 1 p*K* unit deviation from experiment. (c) Comparison of backbone partial charges between Amber14SB and Amber14SB-H. (d) Correlation between calculated and experimental histidine p*K*_a_ values for the modified Amber14SB-H. Error bands and marker color scheme match panel b. (e) Comparative prediction performance across the considered force fields. Consensus is the average of CHARMM36m and Amber14SB-H.

In the course of our coupling analysis, we found that histidine p*K*_a_ values are predicted significantly higher with Amber14SB than with CHARMM36m (Figure 4b). We previously observed lower accuracy for the prediction of lysine p*K*_a_s with the Amber14SB force field: this was traced to the partial charge difference of the backbone between the charged and uncharged lysine species.^17^ We hypothesized that this may also play a role for histidine, as here too the backbone partial charges differ between the doubly (denoted HSP) and singly protonated histidine residues (denoted HSD and HSE). To further investigate, we computed 22 histidine p*K*_a_ values that were taken from a full data set previously probed using equilibrium free energy calculations.^53^ These calculations were performed using both plain Amber14SB and a modified version, here called Amber14SB-H, where the partial charges of the protonated histidine backbone are those previously reported by Best et al (Figure 4c).^54^

To account for the fact that two neutral histidine tautomers can exist with the proton present on *N*δ (HSD) or *N*ϵ (HSE), we perform two sets of free energy calculations: HSP → HSD and HSP → HSE, which yield two relative free energies of deprotonation: ΔΔ*G*_δ_ and ΔΔ*G*_ϵ_. Taking their difference gives the relative free energy of tautomer interconversion:
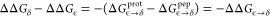
2which we can combine with the absolute free energy of tautomer conversion—determined from the experimental microscopic p*K*_a_ values as (55)—to get the overall relative free energy of deprotonation (Figure 4a):
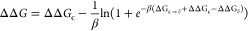
3from which we can determine the p*K*_a_ via eq 1.

Compared to plain Amber14SB, using Amber14SB-H significantly reduced the AUE from 2.06 ± 0.33 p*K* to 0.64 ± 0.11 p*K* and increased the Pearson correlation from 0.65 ± 0.14 to 0.88 ± 0.05 (Figure 4d,e). Unlike previously observed for aspartate, glutamate, and lysine, we found a consensus estimate for CHARMM36m and Amber14SB-H resulted in an predictor that exceeded the performance of either method alone (i.e., AUE: 0.24 ± 0.04 p*K*, Pearson correlation: 0.98 ± 0.01). This level of accuracy exceeded that achieved using FEP+ (i.e., 0.39 p*K*) on the same 22 p*K*_a_ data set (Figure 4e).^53^

Our results suggest that NES can resolve histidine p*K*_a_ values as accurately as FEP+ and further supports our previous suggestion that free energy calculations, in particular p*K*_a_ calculations, with Amber14SB should employ the more recent, Best et al. partial charges.^54^ We note that this partial charge suggestion may also apply to Amber19SB, which utilizes the same backbone charges as Amber14SB.

### Effective Polarization: CHARMM36m-ECC

Traditional MM force fields do not explicitly account for electronic polarizability. While the Drude^56^ and AMOEBA^57^ force fields explicitly introduce this missing electronic polarization, it can also be introduced implicitly. The electronic continuum correction (ECC) models the simulated system as a collection of point charges embedded in a medium.^58,59^ This medium has a dielectric constant of ≈2, corresponding to the high-frequency dielectric of most condensed phase environments.^59^ Applying this as a screening factor into the Coulomb equation effectively scales charges by . In condensed-phase calculations, like the ones considered here, the ECC approximation is reasonable and has been shown to improve thermodynamic and kinetic observables across diverse biomolecular systems.^60−62^ Given that sulfur is significantly more polarizable than oxygen and nitrogen, we hypothesized that the notably poorer p*K*_a_ prediction performance for cysteine—compared to glutamate, aspartate, histidine, and lysine—stems from inaccurately modeled electrostatic interactions between the cysteine thiolate and its protein-residue neighborhood. By reintroducing the missing polarization implicitly, we aimed to refine the representation of this local environment and, in turn, improve the accuracy of our free energy calculations.

**Figure 5:**
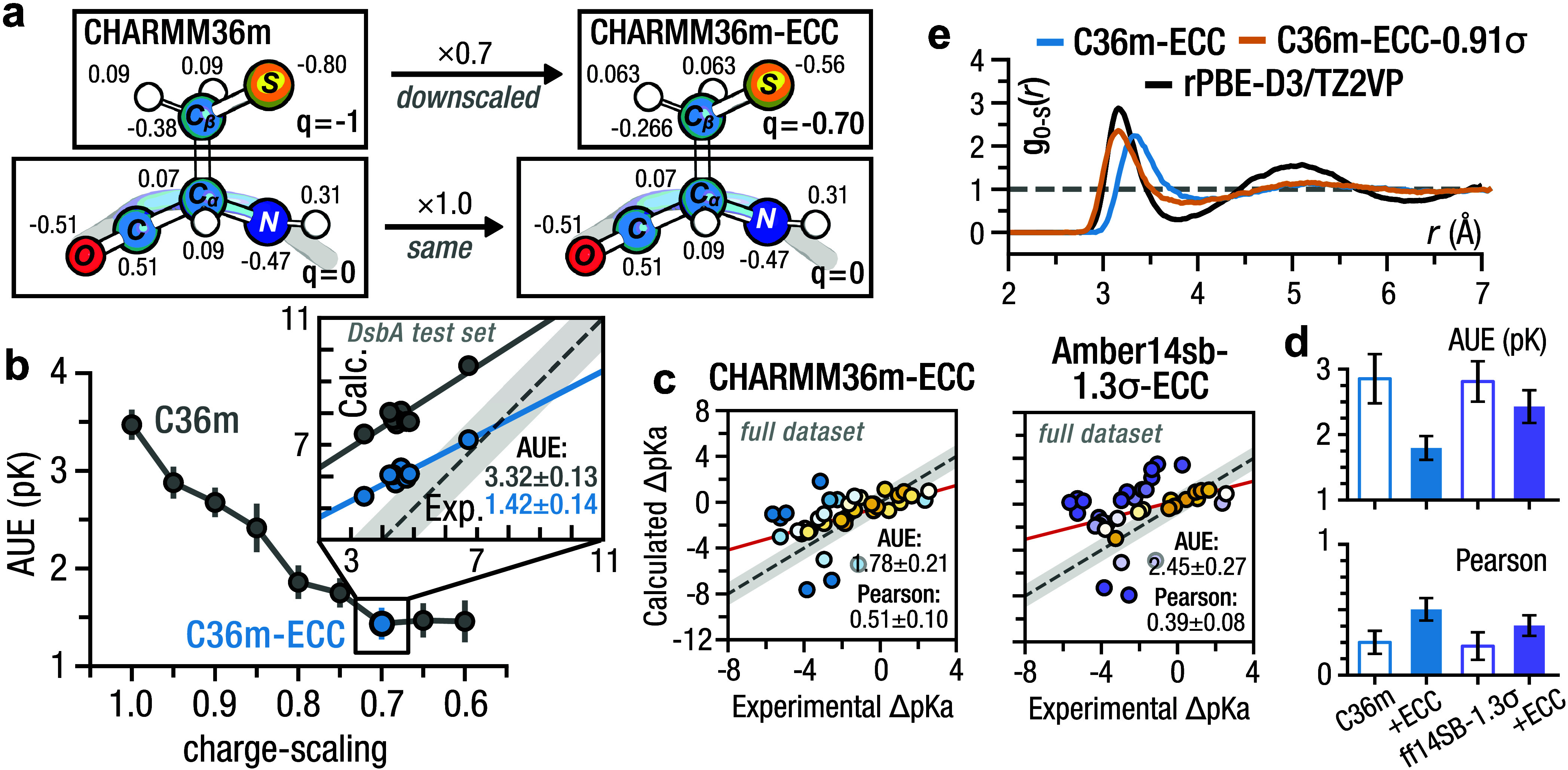
Effective polarization: CHARMM36m-ECC. (a) Charge scaling scheme where the side chain unit charge is scaled while the backbone remains fixed. (b) AUE on the DsbA test set as a function of scaling factor. The value of 0.70 for which the error saturates is marked in blue. (b, inset) Correlation between the calculated and experimental DsbA test set p*K*_a_ values. Regression lines are shown, and the gray error band represents a 1 p*K* unit deviation from experiment. (c) Correlation between the calculated and experimental cysteine p*K*_a_ values. Marker color indicates deviation from experiment where yellow indicates a minimum AUE from experiment. Regression lines are indicated in red, and the gray error band represents a 1 p*K* unit deviation from experiment. (d) Prediction performance comparison between the unscaled (empty bars) and charge-scaled (filled bars) force fields. (e) Solvation structure of charge-scaled methylthiolate with and without σ-scaling on the sulfur atom.

We rescaled all full unit charges (i.e., charged side chains and ions) (Figure 5a) on the interval [0.60, 1.00] with a 0.05 increment in CHARMM36m and recomputed the p*K*_a_ values of the DsbA test set. CHARMM36m was chosen because of its higher accuracy in predicting cysteine p*K*_a_ and because recent charge scaling efforts have successfully employed this force field.^61^

We observed the accuracy saturated to an AUE of 1.42 ± 0.14 p*K* for a scaling of 0.70 (Figure 5b), very close to 0.75, which is the value often used within ECC scaling frameworks (e.g., prosEECo75). Applying this 0.70 scaled CHARMM36m force field on the entire cysteine data set reduced the AUE from 2.92 ± 0.35 p*K* to 1.78 ± 0.21 p*K* and increased the correlation with experiment from 0.24 ± 0.09 to 0.51 ± 0.09 (Figure 5c,d). Accounting for residue coupling improved the accuracy of CHARMM36m-ECC even further, shifting the overall AUE from 1.78 ± 0.21 p*K* to 1.61 ± 0.21 p*K* (Figure S2). Compared to plain CHARMM36m, CHARMM36m-ECC did not significantly improve the already strong histidine p*K*_a_ prediction performance (i.e., 0.69 ± 0.16 p*K* vs 0.71 ± 0.16 p*K*). Charge-scaling did also not completely resolve the significant p*K*_a_ underestimation observed for YopH tyrosine phosphatase (PDB: 1YPT) (Figure S8a). While we could exactly reproduce the relative effects of two nearby mutations (Figure S8c), the absolute p*K*_a_ values were downshifted by ≈4 p*K* units (Figure S8b).

Having scaled down the charge of cysteine, we have also increased the effective radius of the side chain atoms, in particular sulfur. Comparing the solvation structure of charge-scaled methylthiolate to the AIMD simulations, we found a slight increase in the position of the first RDF peak (Figure S9a). Scaling the sulfur σ by 0.91 maximized overlap between with the MD and AIMD RDF curves (Figure 5e, Figure S9b).

As a cross check we also computed solvation free energies of charge- and σ-scaled methylthiolate. Within the ECC framework, absolute free energies cannot be compared directly with experiment but must be adjusted to account for the scaling (see SI methods). After adjusting the values, we found that similar to unscaled CHARMM36m, a slightly larger σ scaling is required to reproduce the experimental solvation free energy (Figure S9b).

Applying the charge-scaled *and σ*-scaled CHARMM36m force field on the 1A2L test set showed no significant improvement (Figure S9c); we did not probe the entire data set with this doubly modified force field.

As an alternative to scaling all unit charges, we charge-scaled only the probed cysteine and balanced the missing negative charge by scaling the ions in solution. Computing the p*K*_a_ values on the DsbA test set revealed a similar improvement trend as observed for global charge scaling, but nevertheless yielded a slightly poorer accuracy at a 0.7 scaling (Figure S11). This difference is quite small and would seem to suggest that charge-scaled interactions of the probed cysteine itself and not that of other charged species is the major determinant of improved accuracy. In certain highly charged contexts (i.e., enzyme active sites), the accuracy improvement from charge-scaling other nearby residues is likely to play a larger role.

As a complete alternative to charge-scaling we also considered the more traditional reparameterization approach of redistributing the charge on the side chain. We found that altering the proportion of charge on the Cβ carbon and sulfur did not improve p*K*_a_ prediction accuracy on the DsbA test set (Figure S10).

Given the success with CHARMM36m, we also investigated charge-scaling with Amber14SB and Amber14SB–1.3σ. Amber presents difficulties because unlike CHARMM the side chain does not carry a full integer charge and cannot be simply scaled. Instead we linearly interpolate between the protonated and deprotonated cysteine to get a charge-scaled residue. Charge scaling Amber14SB improved prediction accuracy on the DsbA test set but failed to meaningfully saturate on the interval [1.00, 0.60], while charge-scaling Amber14SB-1.3σ improved prediction accuracy which was maximized for 0.80 and appeared to degrade for further scaling (Figure S11). Probing Amber14SB-1.3σ with 0.80 charge scaling on the entire data set improved the accuracy from an AUE of 2.88 ± 0.38 p*K* to 2.45 ± 0.27 p*K* (Figure 5c,d); this improvement was smaller than that observed for CHARMM36m. Accounting for coupling also pushed the accuracy slightly higher to 2.37 ± 0.29 p*K*. Compared to plain Amber14SB-H, charge-scaling did improve histidine p*K*_a_ prediction accuracy decreasing the average unsigned error from 0.64 ± 0.11 p*K* to 0.50 ± 0.10 p*K*.

To assess structural effects of charge-scaling on the simulated ensembles (e.g., unfolding) we analyzed the fluctuation profiles of the end-state ensembles. To compare the unscaled and scaled force fields we calculated the absolute difference between the residue-wise RMSF profile: |RMSF_A_ – RMSF_B_|/RMSF_B_ and took the average. This measure varied between proteins but was roughly ≈0.15 Å (Figure S12), which was comparable to the difference observed between unscaled Amber14SB and CHARMM36m. While a comprehensive validation of charge-scaled force fields against their unscaled counterparts is beyond the scope of this work, our observations—along with previous work demonstrating the stability of charge-scaled force fields in larger systems over longer time scales^62^—we suggest that charge-scaling is unlikely to deleteriously destabilize folded protein systems.

Taken as a whole, our results suggest charge scaling is a viable, force field independent method for improving or maintaining the already strong performance of nonpolarizable force fields in p*K*_a_-related, free energy calculations.

### Overall Performance: Modified Force Fields

**Figure 6:**
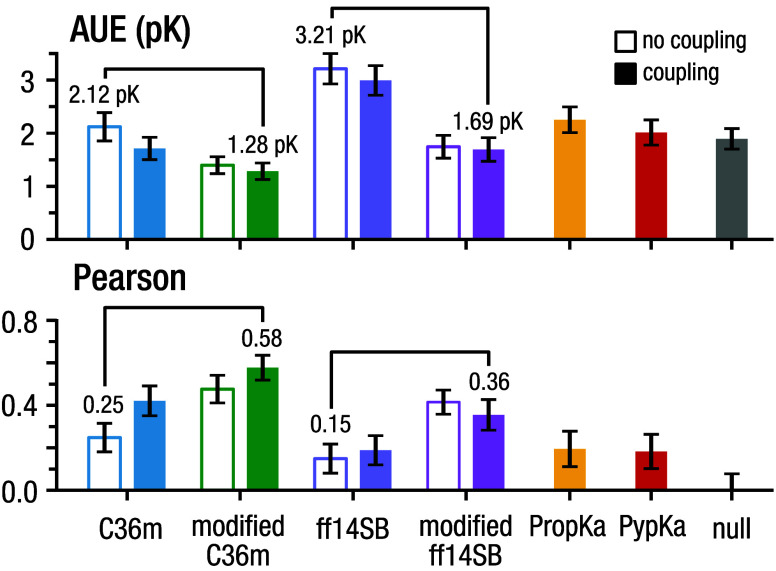
Overall performance: modified force fields. Combined cysteine and histidinep*K*_a_ prediction performance comparison between unmodified and modified CHARMM36m and Amber14SB force fields, with and without coupling accounted for, PropKa, PypKa, and a null model.

Figure 6 summarizes the main findings: on the full dataset our NES approach with the modified CHARMM36m force field and residue coupling accounted for, significantly exceeds the performance of plain CHARMM36m reducing the average unsigned error from 2.12 ± 0.27 p*K* to 1.28 ± 0.15 p*K* and increasing the correlation with experiment from 0.25 ± 0.09 to 0.58 ± 0.08. For Amber14SB, our force field modifications decrease the error from 3.21 ± 0.29 p*K* to 1.69 ± 0.23 p*K* and increase the correlation from 0.15 ± 0.10 to 0.36 ± 0.10. On the same dataset Prop*K*_a_ and Pyp*K*_a_ gave errors of 2.25 ± 0.24 p*K* and 2.02 ± 0.23 p*K*, and correlations of 0.19 ± 0.12 and 0.18 ± 0.12, respectively. The average unsigned error of a null model was 1.89 ± 0.19 p*K.* Considering cysteine predictions within a certain tolerance, CHARMM36m-ECC correctly predicts 39 ± 8% of residues within 1 p*K* and 73 ± 7% within 2 p*K*, compared to 22 ± 6% and 44 ± 8% with Pyp*K*_a_ and 15 ± 6% and 34 ± 7% with Prop*K*_a_. We also note that CHARMM36m-ECC exceeds the AUE of a null model by 0.72 ± 0.21 p*K* or 0.91 ± 0.27 p*K* depending on whether coupling is accounted for.

Considering general determinants of accuracy we observed—consistent with previous work^17^—that the p*K*_a_ itself is a reasonable predictor of accuracy: performance degraded as a function of p*K*_a_ (Figure S13a). Decreasing p*K*_a_ also correlated with solvent exposure i.e., buried residues tended to have lower p*K*_a_ values (Figure S13b) and, by extension, solvent accessibility correlated with the prediction error (Figure S13c). In this data set, buried residues are also more frequently involved in coupling (Figure S13c, triangle markers). As noted earlier, coupling is an important determinant of accuracy, with poorer prediction performance observed for coupled residues compared to uncoupled ones. As also noted earlier, this discrepancy can be remedied by explicitly accounting for coupling, which restores the accuracy to a level comparable to that observed for uncoupled residues (Figure S2).

Taken together, this final comparison highlights that NES p*K*_a_ prediction accuracy can be significantly increased by accounting for charge scaling and residue coupling. These enhancements push the accuracy well above unscaled force fields and conventional predictor methods.

## Discussion

Here, we assess the ability of nonequilibrium switching (NES) free energy calculations to resolve the p*K*_a_ values of 40 cysteine and 22 histidine residues across 10 wildtype and mutant proteins. Given the widespread use of free energy calculations in lead optimization and the growing interest in designing targeted covalent inhibitors, *in silico* methods for determining the deprotonation free energy of specific cysteines in the presence or absence of bound molecules is highly desirable.

Our results highlight three force field modifications that can improve p*K*_a_ prediction accuracy for cysteine and histidine: (1) increasing the vdW radius of the deprotonated cysteine sulfur in Amber14SB; (2) altering the backbone partial charges of doubly protonated histidine in Amber14SB; and (3) charge scaling all unit charges in CHARMM36m and Amber14SB. Our investigation is not intended to provide definitive parameters for either force field or an absolute strategy for improving relative free energy calculations, particularly p*K*_a_ prediction, instead, we aim to highlight potential avenues for further investigation and development.

On the full data set of 40 cysteines and 22 histidines, we found the strongest performing force field, CHARMM36m-ECC, to exhibit an AUE of 1.61 ± 0.21 p*K* for cysteine; this accuracy exceeds conventional predictors and a null model. While increasing the vdW of sulfur and charge-scaling both improved the performance of Amber14SB in predicting cysteine p*K*_a_ values, the final accuracy of 2.36 ± 0.29 p*K* remains lower than that of CHARMM36m, suggesting further reparameterization of the residue would be required.

In the case of histidine, we found the accuracy could be significantly improved by taking a consensus of the Amber14SB-H and CHARMM36m charge-scaled force fields which yielded an AUE of 0.24 ± 0.04 p*K* and a Pearson correlation of 0.98 ± 0.01. Even standing alone, Amber14SB-H with charge-scaling attained an accuracy of 0.50 ± 0.10 p*K* and correlation of 0.85 ± 0.06, while CHARMM36m with charge-scaling gave an accuracy of 0.71 ± 0.16 p*K* and correlation of 0.56 ± 0.15.

We note that while NES paired with the charged-scaled CHARMM36m force field represents the strongest predictor reported here, our results suggest inherent limitations of conventional force fields in accurately capturing the local electrostatic environment of the cysteine thiolate. The deprotonated sulfur is significantly more polarizable than oxygen or nitrogen, making p*K*_a_ predictions particularly sensitive to local electrostatics, hydrogen bonding, and screening effects within the protein. These factors are not fully accounted for and likely explain why prediction accuracy is poorer compared to the less polarizable amino acids i.e., aspartate and lysine. In light of this, alternative approaches may be necessary to improve physics-based cysteine p*K*_a_ predictions. One potential avenue is the use of explicitly polarizable force fields^56,57^ which could offer a more accurate description of electrostatics compared to the implicitly polarizable force fields employed here. Another potential direction is the integration of MM/ML end-state corrections.^63,64^ As machine learning potentials become more capable of reliably modeling charged species at longer ranges, they could be used to correct the solvent and protein branches of the thermodynamic cycle in Figure 1, ultimately leading to more accurate p*K*_a_ estimates.

In summary we find MD-approaches, including NES, can resolve the p*K*_a_ values of cysteine and histidine residues with accuracy that exceeds conventional methods; however, this requires modification to the underlying MD force fields. The largest accuracy improvement we observed was for charge scaling the CHARMM36m force field, a result that will likely extend to other force fields and could remedy the poorer accuracy previously observed for predicting the effect of charge-changing mutations on protein thermostability^65^ and binding affinity.^66^ More work will help determine the consequences of charge-scaling; however, this work and the recent work of others^61,62^ seems to suggest that charge-scaling may be a general method to enhance the accuracy of nonpolarizable MM force fields with only minimal and predictable costs.

## Data Availability

Calculated p*K*_a_ values and modified force field files are available at https://github.com/deGrootLab/pka_reparam_2025.

## Supporting Information Available

A derivation of the ECC solvation free energy relationship, supplementary figures, and supplemental tables, which contain references to PDB structures^67−86^ and experimental p*K*_a_ values^4,5,87−102^ (PDF)
