# Supplementary material for: Improving pKa Predictions with Reparameterized Force Fields and Free Energy Calculations
Source: J Chem Theory Comput. 2025 Apr 2;21(8):4095–106. doi: 10.1021/acs.jctc.5c00031 (PMC12020373; doi:10.1021/acs.jctc.5c00031)
Supplement: Supplementary file 1 — ct5c00031_si_001.pdf [file ct5c00031_si_001.pdf]

Supporting Information: Improving pKa Predictions with Reparameterized Force Fields and Free Energy Calculations

Carter J. Wilson,[†] Vytautas Gapsys,[*,†,‡] and Bert L. de Groot[*,†]

†*Computational Biomolecular Dynamics Group, Max Planck Institute for Multidisciplinary Sciences, 37077 Göttingen, Germany*

‡*Computational Chemistry, Janssen Research & Development, Janssen Pharmaceutica N. V., Turnhoutseweg 30, B-2340 Beerse, Belgium.*

E-mail: vgapsys@gwdg.de; bgroot@gwdg.de

Supplemental Methods

Within the ECC framework the absolute solvation free energies calculated for the charge-scaled methythiolate cannot be directly compared with the experimental value. This is because the energy that arises from electronic polarization (E_{ele}) is removed from the force field (E_{ff}) and treated in a mean field way. The result is the free energy is split according to:

$$\Delta G_{exp} = \Delta G_{nuc} + \Delta G_{elec} = \Delta G_{MD} + \Delta G_{elec}. \tag{S1}$$

If we assume that the medium between MD particles has a relative dielectric constant ϵ_r then ΔG_{ele} can be approximated with a Born model:

$$\Delta G_{Born} = -\frac{N_A z^2 e^2}{8\pi\epsilon_0 r_0}\left(1 - \frac{1}{\epsilon_r}\right) \tag{S2}$$

where N_A, z, e, ϵ_0, and r_0, are Avogadro's constant, the ion charge, the elementary charge, the dielectric constant of vacuum, and the Born radius of the ion.

If we take a scaling factor $q = \frac{1}{\sqrt{e_r}}$ then Equation 2 becomes:

$$\Delta G_{ele} = -\frac{N_A z^2 e^2}{8\pi\epsilon_0 r_0}\left(1 - q^2\right). \tag{S3}$$

We can also approximate the experimental solvation free energy using a Born model

$$\Delta G_{exp} = -\frac{N_A z^2 e^2}{8\pi\epsilon_0 r_0}\left(1 - \frac{1}{\epsilon_{water}}\right) \tag{S4}$$

and observing that $\epsilon_{water} \approx 78 >> 1$ we have

$$\Delta G_{exp} \approx -\frac{N_A z^2 e^2}{8\pi\epsilon_0 r_0}. \tag{S5}$$

Returning to Equation 1 we then have

$$\begin{aligned}
\Delta G_{MD} &= \Delta G_{exp} - \Delta G_{elec} \\
&\approx -\frac{N_A z^2 e^2}{8\pi\epsilon_0 r_0} + \frac{N_A z^2 e^2}{8\pi\epsilon_0 r_0}\left(1 - q^2\right) \\
\Delta G_{MD} &= \Delta G_{exp} \cdot q^2
\end{aligned}$$

Supplemental Tables

Table S1: Table of Experimental cysteine pKa values and PDBs with DOI links.

PDBID	Residue	pKa	pKa Citation	PDB Citation
1QLP	C210	6.9	10.1074/jbc.M203089200	10.1110/ps.9.7.1274
4MA9	C46	5.9	10.1021/bi801718d	10.1021/bi4011573
1THE	C29	3.6	10.1016/S0021-9258(18)42891-8	10.1074/jbc.270.10.5527
1P5F	C106	5.4	10.1021/bi800282d	10.1073/pnas.1133288100
1I0E	C283	5.6	10.1021/bi011208f	10.1107/s0907444901007703
$1I0E_{S285A}$	C283	6.7	10.1021/bi011208f	10.1107/s0907444901007703
1QKI	C278	5.6	10.1021/bi011208f	10.1016/S0969-2126(00)00104-0
2L90	C51	7.2	10.1074/jbc.M112.369116	10.1074/jbc.M112.368936
$2L90_{E115Q}$	C51	8.2	10.1074/jbc.M112.369116	10.1074/jbc.M112.368936
1EH6	C145	5.3	10.1021/bi034937z	10.1093/emboj/19.7.1719
1PPN	C25	3.3	10.1021/bi9705974	10.1107/S0108768191006572
1PPO	C25	2.9	10.1021/bi9705974	10.1107/S0108768191003191
1ERT	C32	6.3	10.1021/bi00128a019	10.1016/S0969-2126(96)00079-2
1XOB	C32	7.3	10.1021/bi970071j	10.1016/s0969-2126(94)00086-7
1O73	C40	7.2	10.1074/jbc.RA118.006366	10.1074/jbc.M301526200
1SU9	C35	8.8	10.1074/jbc.M607047200	10.1074/jbc.M402823200
2HNP	C215	5.6	10.1021/bi963094r	10.1126/science.8128219
1JAS	C88	10.2	10.1021/bi0514459	10.1023/A:1013807519703
1JBB	C85	11.1	10.1021/bi0514459	10.1023/A:1013807519703
1I7K	C85	10.9	10.1021/bi0514459	10.1074/jbc.M109398200
1YPT	C213	4.7	10.1021/bi00087a012	10.1038/370571a0
$1YPT_{H402N}$	C213	5.99	10.1021/bi00087a012	10.1038/370571a0
$1YPT_{H402A}$	C213	7.35	10.1021/bi00087a012	10.1038/370571a0
1A2L	C28	3.3	10.1016/0092-8674(95)90210-4	10.1016/s0969-2126(98)00077-x
$1A2L_{LT}$	C28	4.86	10.1016/0092-8674(95)90210-4	10.1016/s0969-2126(98)00077-x
$1A2L_{PL}$	C28	4.42	10.1016/0092-8674(95)90210-4	10.1016/s0969-2126(98)00077-x
$1A2L_{PP}$	C28	6.73	10.1016/0092-8674(95)90210-4	10.1016/s0969-2126(98)00077-x
$1A2L_{QL}$	C28	4.59	10.1016/0092-8674(95)90210-4	10.1016/s0969-2126(98)00077-x
$1A2L_{SF}$	C28	4.34	10.1016/0092-8674(95)90210-4	10.1016/s0969-2126(98)00077-x
$1A2L_{ST}$	C28	4.45	10.1016/0092-8674(95)90210-4	10.1016/s0969-2126(98)00077-x
$1A2L_{PL}$	C28	4.23	10.1016/0092-8674(95)90210-4	10.1016/s0969-2126(98)00077-x
$1A2L_{TR}$	C28	4.76	10.1016/0092-8674(95)90210-4	10.1016/s0969-2126(98)00077-x
$1NTI_{E78C}$	C78	9.6	10.1021/bi4016633	N/A
$1NTI_{S65C}$	C65	9.0	10.1021/bi4016633	N/A
$1NTI_{M46C}$	C46	8.2	10.1021/bi4016633	N/A
$1NTI_{V36C}$	C36	9.5	10.1021/bi4016633	N/A
$1NTI_{T17C}$	C17	9.8	10.1021/bi4016633	N/A
$2MGE_{G124C}$	C125	6.5	10.1110/ps.0224203	10.1006/jmbi.1993.1569
$2MGE_{A125C}$	C126	8.4	10.1110/ps.0224203	10.1006/jmbi.1993.1569
$2MGE_{D126C}$	C127	8.1	10.1110/ps.0224203	10.1006/jmbi.1993.1569

Table S2: Table of Experimental histidine pKa values and PDBs with DOI links.

PDBID	Residue	pKa	pKa Citation	PDB Citation
1A6K	H12	6.49	10.1016/S0006-3495(00)76414-9	10.1016/S0006-3495(99)77056-6
1A6K	H36	7.98	10.1016/S0006-3495(00)76414-9	10.1016/S0006-3495(99)77056-6
1A6K	H48	5.55	10.1016/S0006-3495(00)76414-9	10.1016/S0006-3495(99)77056-6
1A6K	H81	6.88	10.1016/S0006-3495(00)76414-9	10.1016/S0006-3495(99)77056-6
1A6K	H113	5.51	10.1016/S0006-3495(00)76414-9	10.1016/S0006-3495(99)77056-6
1A6K	H116	6.70	10.1016/S0006-3495(00)76414-9	10.1016/S0006-3495(99)77056-6
1DWR	H36	7.8	10.1016/S0006-3495(00)76414-9	10.1038/35002641
1DWR	H81	6.94	10.1016/S0006-3495(00)76414-9	10.1038/35002641
1DWR	H113	5.87	10.1016/S0006-3495(00)76414-9	10.1038/35002641
1DWR	H116	6.79	10.1016/S0006-3495(00)76414-9	10.1038/35002641
1DWR	H119	6.56	10.1016/S0006-3495(00)76414-9	10.1038/35002641
1ERT	H43	5.5	10.1021/bi00128a019	10.1016/s0969-2126(96)00079-2
1EY0	H8	6.52	10.1021/bi0119417	10.1006/jmbi.2000.4140
1EY0	H46	5.86	10.1021/bi0119417	10.1006/jmbi.2000.4140
1EY0	H121	5.86	10.1021/bi0119417	10.1006/jmbi.2000.4140
1EY0	H124	5.86	10.1021/bi0119417	10.1006/jmbi.2000.4140
2LZM	H31	9.1	10.1021/bi00461a025	10.1016/0022-2836(87)90636-x
2RN2	H62	7.0	See reference below	10.1016/0022-2836(92)90260-q
2RN2	H83	5.5	See reference below	10.1016/0022-2836(92)90260-q
2RN2	H124	7.1	See reference below	10.1016/0022-2836(92)90260-q
2RN2	H127	7.9	See reference below	10.1016/0022-2836(92)90260-q

Reference: Role of Histidine 124 in the Catalytic Function of Ribonuclease HI from Escherichia coli, Oda, Y. et al., *Journal of Biological Chemistry* 268(1) 88-92, 1993

Supplemental Figures

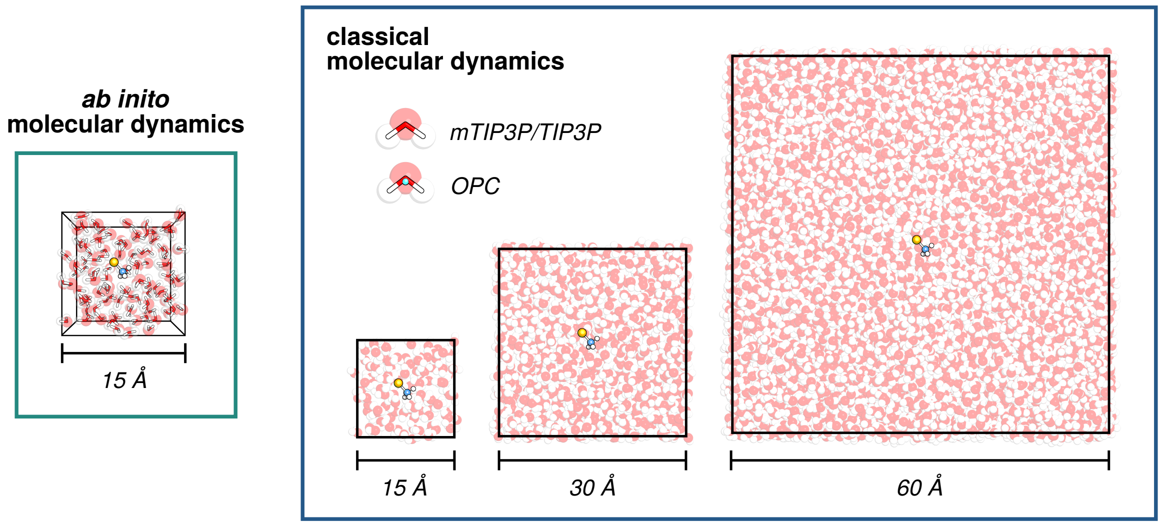

Figure S1: **System setups used for methylthiolate calculations.** The *ab initio* (AIMD) box was 15 Å in size with 109 water molecules and a single methylthiolate. Three boxes were used for classical simulations: 15 Å, 30 Å, and 60 Å. The 30 Å box was the primary box used for calculations; the 15 Å box was used to determine whether there was a significant box size effect on the radial distribution function calculated from the AIMD trajectory; and the 60 Å was used to determine any box size effect on the charge-changing solvation free energy calculations. For CHARMM36m two water models were also assessed for their effect on the calculated thermodynamic properties.

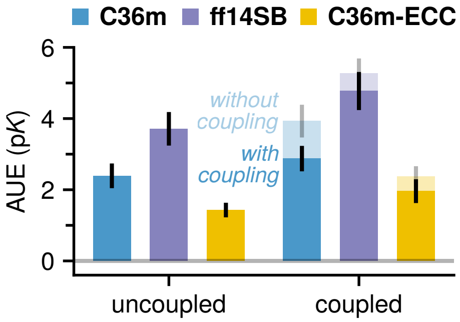

Figure S2: **Residue coupling.** We observe lower prediction accuracy for coupled residues as compared to uncoupled residues. For coupled residues, solid bars correspond to predictions made with coupling accounted for, while transparent correspond to predictions made without coupling accounted for.

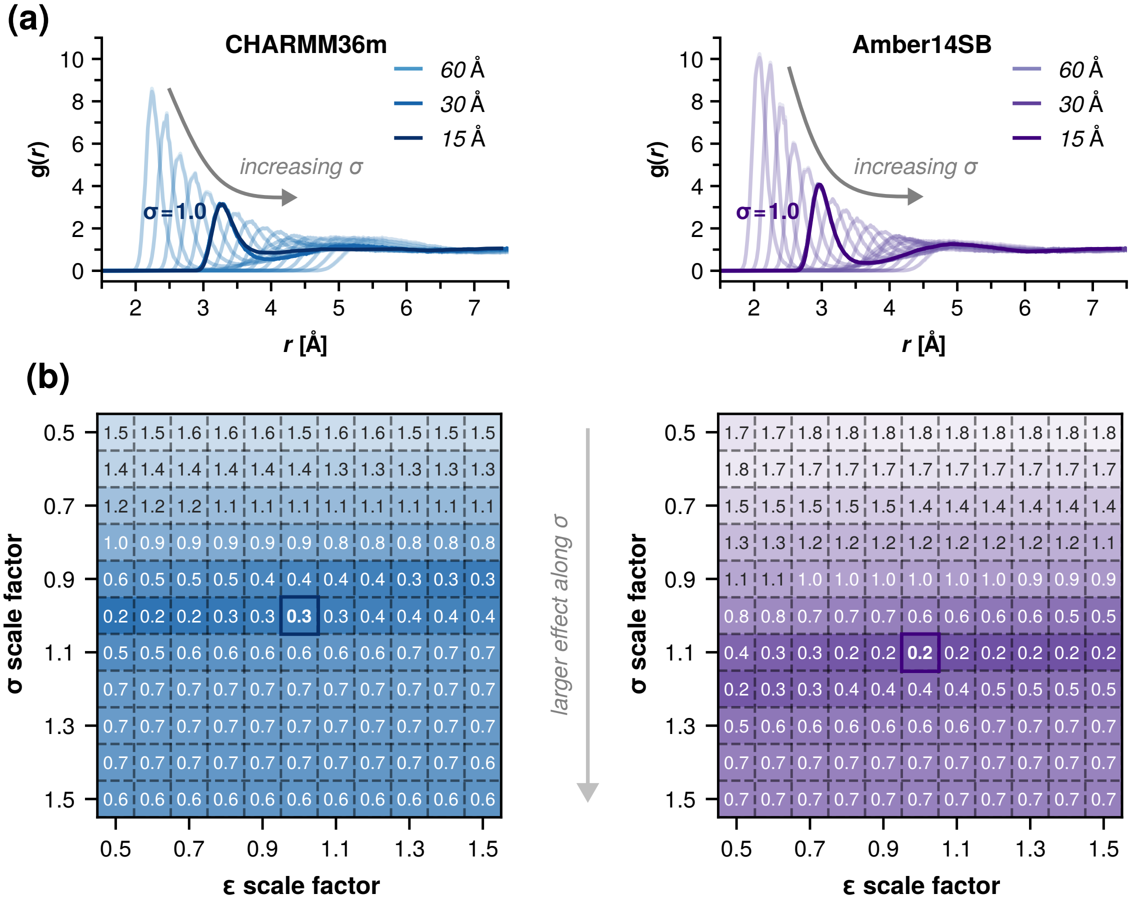

Figure S3: **Radial distribution functions of methylthiolate.** **(a)** We observe no qualitative difference when using a different box size for calculating the radial distribution function (RDF) for methylthiolate; the same shift in the first peak as a function of increasing methylthiolate sulfur σ is observed. Note that the significant overlap between curves makes distinguishing them difficult. **(b)** Exploring the RDF RMSD matrix of (σ, ϵ) pairs reveals that changes to σ alter the value significantly more than changes in ϵ. For CHARMM36m, the default value of $\sigma = 1.00$ well reproduces the position of the first RDF peak calculated from *ab initio* molecular dynamics, while for Amber14SB a value of $\sigma \approx 1.10$ is required. The (σ, ϵ) pair that minimizes the RMSD between the MD and AIMD RDF curves is indicated with a box.

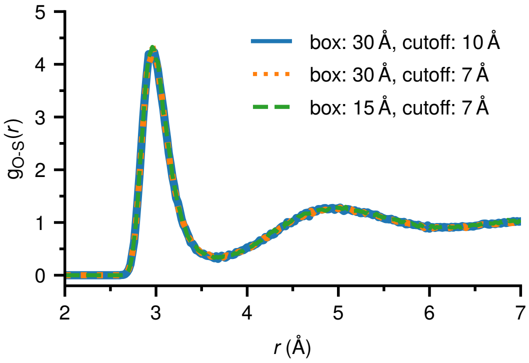

Figure S4: **Radial distribution functions of methylthiolate.** We observe no qualitative difference on the solvation structure when using a 7 AA cutoff in a s = 15Å box, a 7 Å cutoff scheme in a s = 30Å box, or a 10Å cutoff in a s = 30 Å box. Note that lines overlap each other.

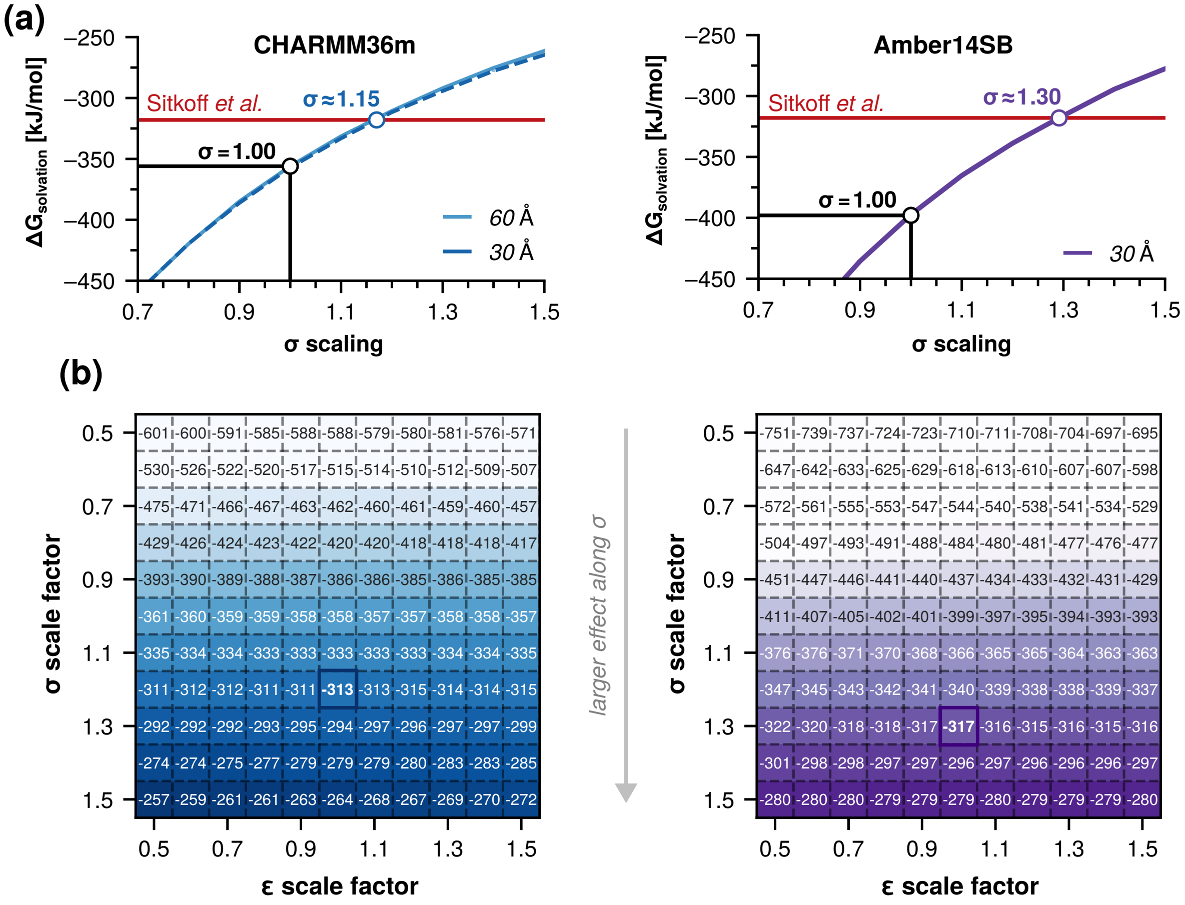

Figure S5: **Solvation free energy of methylthiolate.** **(a)** We observe no difference when using a different box size for calculating the solvation free energy of methylthiolate; the same methylthiolate sulfur σ dependence is observed in both cases. For CHARMM36m, a value of $\sigma \approx 1.15$ reproduces the experimental value (red line), while in the case of Amber14SB a value of $\sigma \approx 1.30$ is required. **(b)** Exploring the solvation free energy matrix of (σ, ϵ) pairs reveals that changes to σ alter the value significantly more than changes in ϵ. The (σ, ϵ) pair that reproduces the experimental value of –318 kJ/mol is indicated with a box, in the case of CHARMM36m this sits between two σ values.

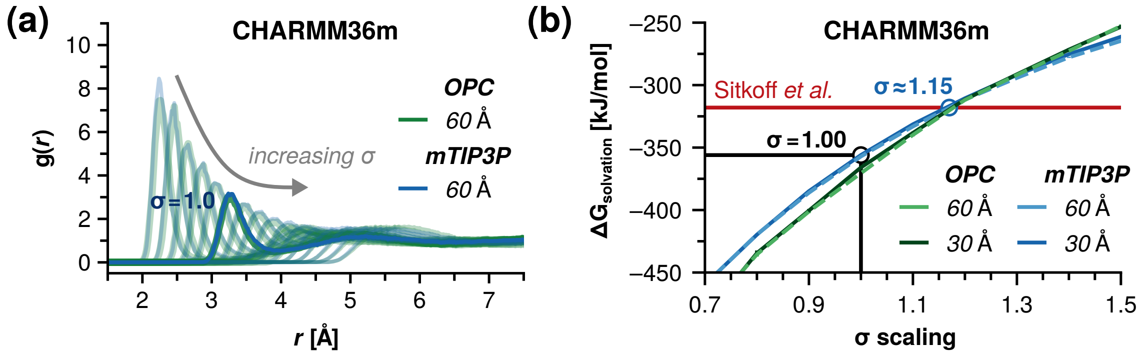

Figure S6: **Water model and methylthiolate properties.** **(a)** In the 60 Å box we observe no qualitative difference in the RDF structure when using a 4-point (OPC, green) or 3-point (modified TIP3P, blue) water model. Increasing σ shifts the position of the first peak by the same amount for both models. **(b)** Slightly different slopes are observed for the solvation free energy dependence on σ when using OPC (green) or mTIP3P (blue). The difference is larger than that observed for using a smaller box (e.g. 30 Å). Slightly different solvation free energies are calculated for the default sulfur thiolate: σ = 1.00; however, both models would suggest an identical sulfur thiolate: $\sigma \approx 1.15$, for reproducing the experimental solvation free energy of methylthiolate (red line).

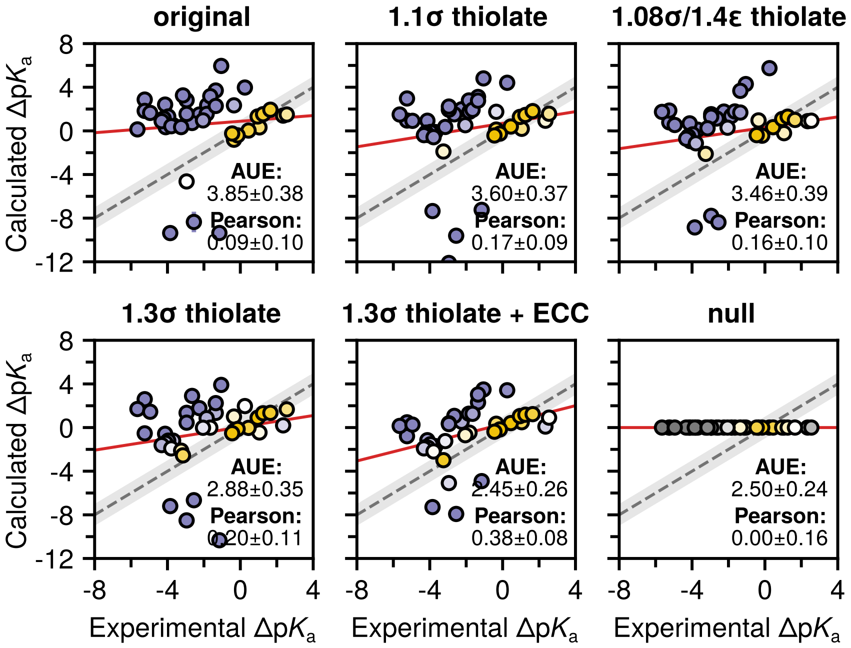

Figure S7: **Performance of Amber14SB variants.** The original Amber14SB force field is compared with variants that scale the cysteine thiolate in difference ways: 1.1σ-scaled, the 1.08σ/1.4ϵ-scaled, 1.3σ-scaled, and 1.3σ-scaled with charge-scaling to 0.8. The 1.08σ/1.4ϵ-scaled parameters were determined based on a fit to the AIMD determined methylthiolate structure.[?]

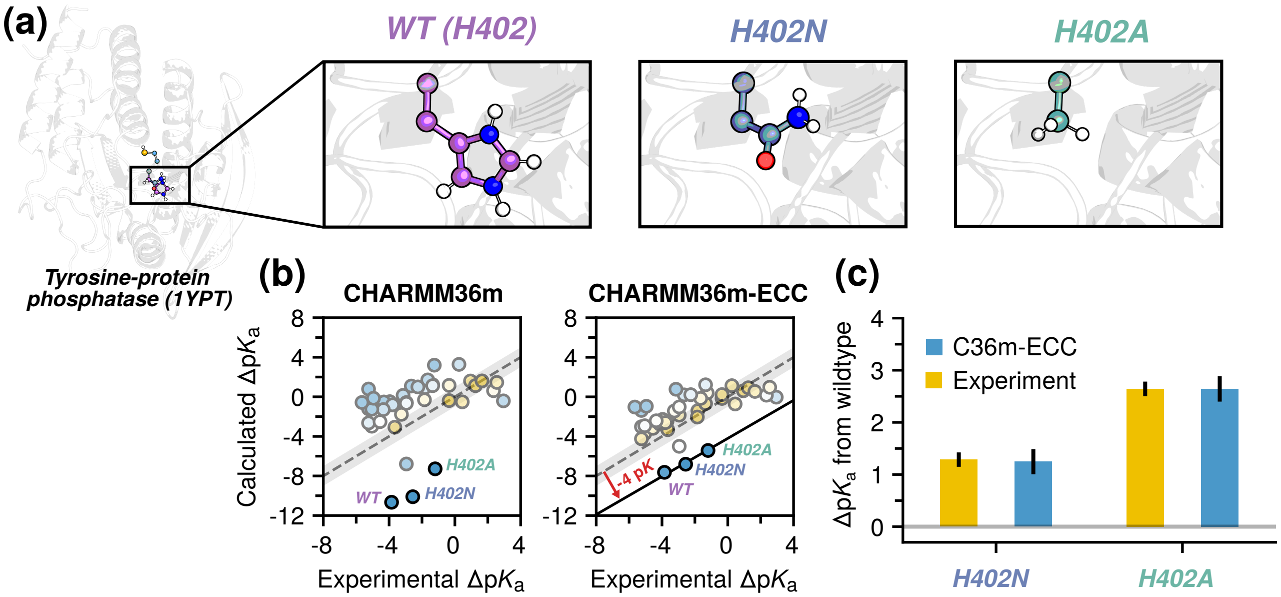

Figure S8: **Yersinia protein tyrosine phosphatase pK_a prediction.** **(a)** The structure of wildtype Yersinia protein tyrosine phosphatase (YopH; PDB:1YPT) and two variants, H402N and H402A, are depicted; these mutations raise the apparent pK_a of cysteine 403. **(b)** The three YopH pK_a values are indicated alongside the remaing 40 calculated pK_a using plain CHARMM36m and CHARMM36m-ECC; note the negative absolute pK_a values (i.e., $\Delta pK_a < -8.55$) calculated from the free energy calculations for plain CHARMM36m. CHARMM36m-ECC improves prediction accuracy; however, a 4 pK downshift remains. **(c)** Calculating the effect of mutation on the pK_a shift relative to wild type we get exact agreement with experiment using CHARMM36m-ECC.

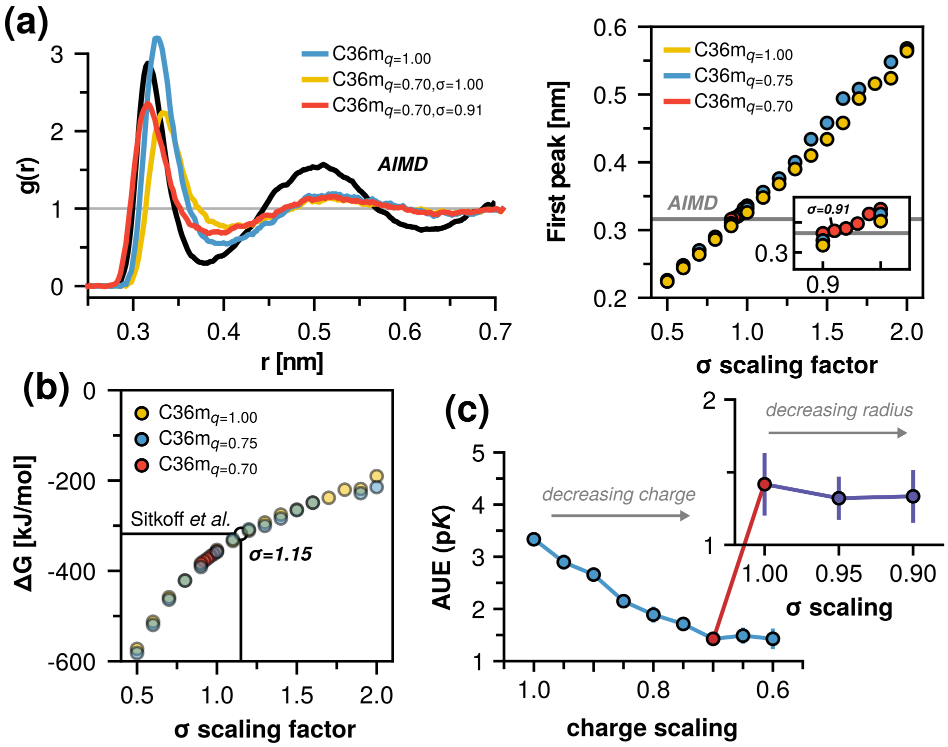

Figure S9: **Scaling σ in charge-scaled CHARMM36m. (a)** Atomistic and AIMD computed RDFs are indicated (left). The position of the first peak in the atomisitc RDF is plotted for different σ-scaling factors (right). Inset shows a zoom over the critical region where a value of $\sigma = 0.91$ gives agreement with the position of the first peak and gives the maximum overlap with the entire first peak (i.e., r $\in$ [0.28, 0.35]) according to $\xi = (\text{MD} - \text{AIMD})^2/\text{AIMD}$ **(b)** Scaled solvation free energies for different charge-scaling and σ-scaling are shown alongside the experimental value; in all cases a larger σ is required to reproduce this value. **(c)** AUE on the DsbA test set as a function of charge-scaling factor (blue) and σ-scaling. In the case of σ-scaling we start with the optimal charge-scaled force field (i.e., 0.70, red point). The effect of reducing the radius of the deprotonated sulfur (i.e., downscaling σ) on the AUE is not significant.

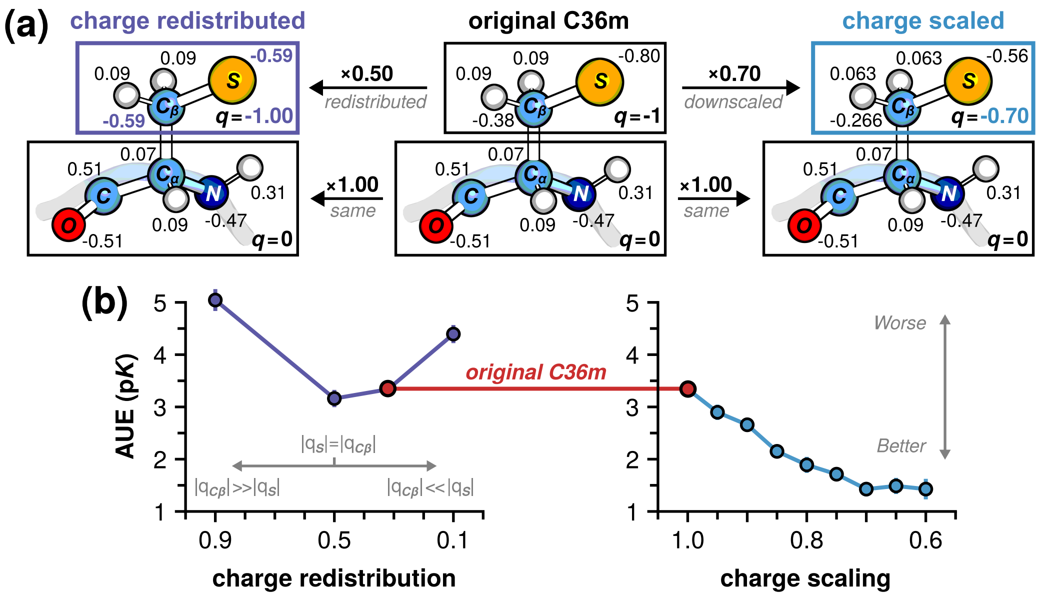

Figure S10: **Charge-scaling versus charge-redistribution.** **(a)** Two schemes for altering the partial charges are shown: 1) charge redistribution where the total side chain charge is preserved but simply rebalanced between heavy atoms and 2) charge-scaling where the side chain charge is downscaled. **(b)** AUE on the DsbA test set as a function of charge-scaling or charge-redistribution factor. Unlike charge-scaling, charge-redistribution does not significantly improve accuracy.

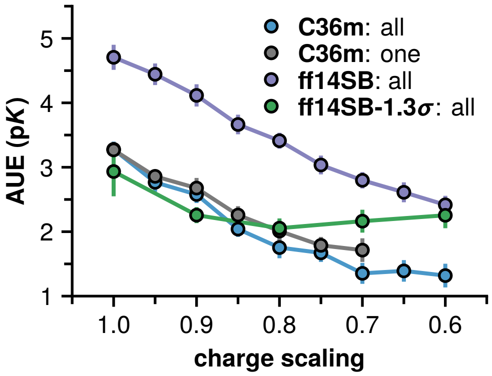

Figure S11: **Charge-scaling schemes and force fields.** AUE on the DsbA test set as a function of charge-scaling factor. For CHARMM36m we consider: 1) a normal charge-scaling scheme where all integer charges are scaled (blue line) and 2) a scheme where only the probed cysteine is scaled and the missing charge is redistributed on the ions in solution. For Amber14SB and Amber14SB-1.3σ we employ the normal scheme of scaling all integer charges.

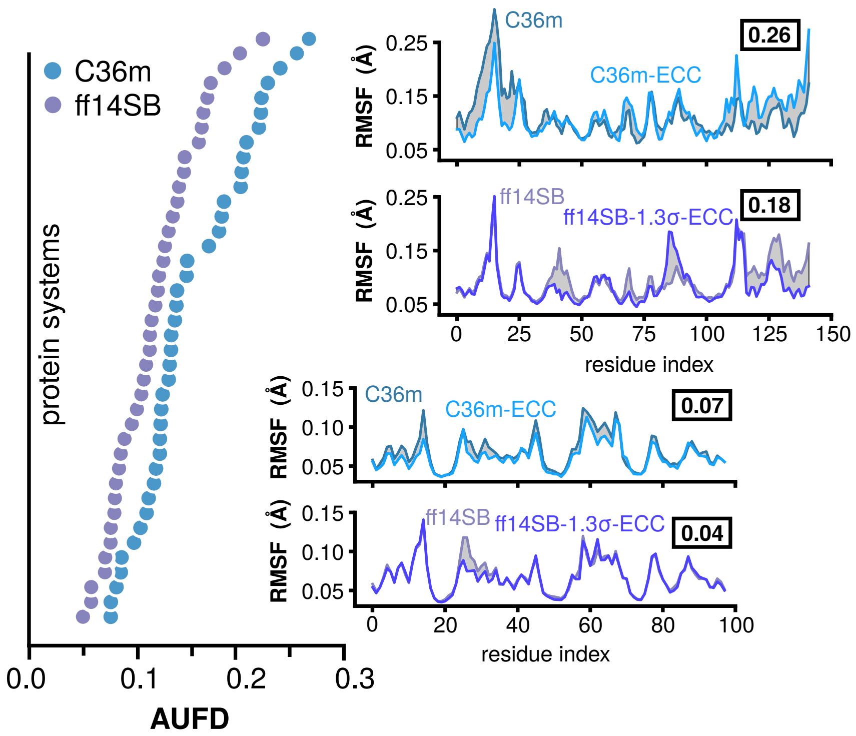

Figure S12: **Protein fluctuations due to charge- and σ-scaling** We calculate the average unsigned fluctuation deviation (AUFD) between plain CHARMM36m and Amber14SB and their scaled counterparts i.e., CHARMM36m-ECC and Amber14SB-1.3σ-ECC. The AUFD is the residue-wise average RMSF: $\langle|\mathrm{RMSF}_A - \mathrm{RMSF}_B|/\mathrm{RMSF}_B\rangle_{\mathrm{residue}}$. Inset plots depict the residue-wise RMSF for the plain and scaled force fields. The value in the upper right is the AUFD.

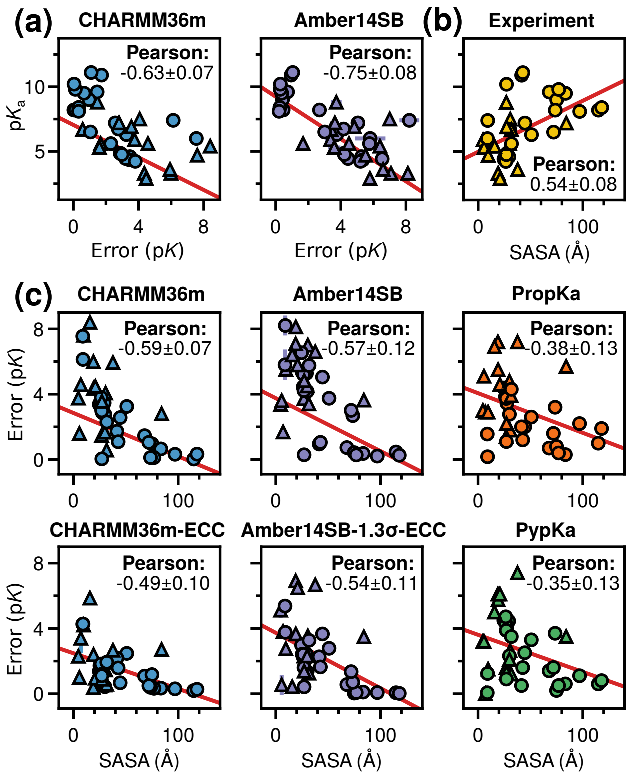

Figure S13: **Prediction error correlations (a)** The pK_a prediction error correlates with the experimental pK_a and **(b)** the experimental pK_a correlates with the solvent accessibile surface area. **(c)** Comparing the error and solvent accesibleity reveals a correlation for all methods. In this dataset, residues identified as coupled (triangle markers) are more likely to be buried (e.g., lower SASA) and exhibit higher prediction error.
